# Electrochemical Enzyme Sensor Based on the Two-Dimensional Metal–Organic Layers Supported Horseradish Peroxidase

**DOI:** 10.3390/molecules27238599

**Published:** 2022-12-06

**Authors:** Yu Xiong, Chao Wang, YuanFei Wu, Chunhua Luo, Dongping Zhan, Shizhen Wang

**Affiliations:** 1 College of Chemistry and Chemical Engineering, Xiamen University, Xiamen 361005, China; 2 The First College of Clinical Medical Science, China Three Gorges University, Yichang 443003, China

**Keywords:** electrochemical enzyme sensor, metal–organic layers, horseradish peroxidase, hydrogen peroxide

## Abstract

Metal–organic frames (MOFs) have recently been used to support redox enzymes for highly sensitive and selective chemical sensors for small biomolecules such as oxygen (O_2_), hydrogen peroxide (H_2_O_2_), etc. However, most MOFs are insulative and their three-dimensional (3D) porous structures hinder the electron transfer pathway between the current collector and the redox enzyme molecules. In order to facilitate electron transfer, here we adopt two-dimensional (2D) metal–organic layers (MOLs) to support the HRP molecules in the detection of H_2_O_2_. The correlation between the current response and the H_2_O_2_ concentration presents a linear range from 7.5 μM to 1500 μM with a detection limit of 0.87 μM (S/N = 3). The sensitivity, reproducibility, and stability of the enzyme sensor are promoted due to the facilitated electron transfer.

## 1. Introduction

HRP-based electrochemical biosensors are of great importance for the rapid, accurate, and reliable detection of H_2_O_2_ in medical diagnosis as well as in food, pharmacy, and environment analysis [1,2,3]. Because the redox center of HRP is embedded in the hydrophobic amino acid residues, the electron transfer pathway between the HRP and the electrode is crucial for the high level of activity and selectivity of the biosensor. Chemically modified electrodes (CMEs) were proposed to solve this problem by optimizing the molecular orientations of HRP on CMEs by the surface charge or nanostructures as well as by facilitating the conductive network of electron transfer [4,5]. For example, the protonated amino groups are usually grafted on the CMEs surface to provide the electrostatic microenvironment for the molecular orientation of HRP since it is overall negatively charged [6]; and the metal nanoparticles [7], carbon nanotubes [8], and graphene [9,10] were adopted as current collectors to form the electron transfer network.

Three-dimensional porous materials have been widely used as enzyme immobilization carriers due to their tunable structure, high specific surface area, and high biocompatibility, including kaolin [11], zeolites [12], MOFs [13], etc. MOFs are widely used for sensors due to their large surface area, adjustable pore size, and functional sites. Li et al. highlighted the most recent progress in MOF for the sensing and switching materials in the detection of gases and volatile organic compounds [14]. Koo et al. reviewed the current application of pure MOFs, MOF membranes, and MOF derivatives as chemiresistors, as well as the future development of MOFs [15]. Fan et al. reported a [(ZIF-8@HRP/GO)/(GO-PEI)]_4_/ITO biosensor, and the current–concentration response was linear in the range of [20, 6000] μM with a detection limit of 3.4 μM (S/N = 3) [16]. Employing ZIF-67(Co), Liu et al. reported a linear current–concentration response region of [1.86, 1050] μM and a sensitivity of 315 μA·mM^−1^·cm^−2^ [17]. With a NH_2_-MIL-53(Fe)/HRP/MWNTs/GCE, Jiang et al. found two linear current–concentration response ranges of [0.1, 1] μM and [1, 600] μM with a detection limit of 0.028 μM [2]. However, these materials are generally of poor conductivity for electron transfer and, moreover, their surface stacking limits the mass transfer of the substrate, e.g., H_2_O_2_ for HRP [18].

A two-dimensional metal–organic framework has an ultrathin layer structure, which increases the exposed active sites, reduces the resistance and, ultimately, improves its catalytic performance. It is widely used in water splitting reactions, the electro-oxidation of organic molecules, etc. [19]. Here, we adopted a novel ordered 2D material, termed as metal–organic layers (MOLs), NH_2_-Hf-BTB-MOL, as the supporter of HRP molecules to construct the electrochemical H_2_O_2_ sensor. The results show that the 2D MOL-based sensor cannot only facilitate the electron transfer by shortening the transfer distance, but also improve the effective loading amount of HRP, demonstrating the prospective applications of 2D MOLs in electrochemical enzyme sensors.

## 2. Results and Discussions

**Figure 1:**
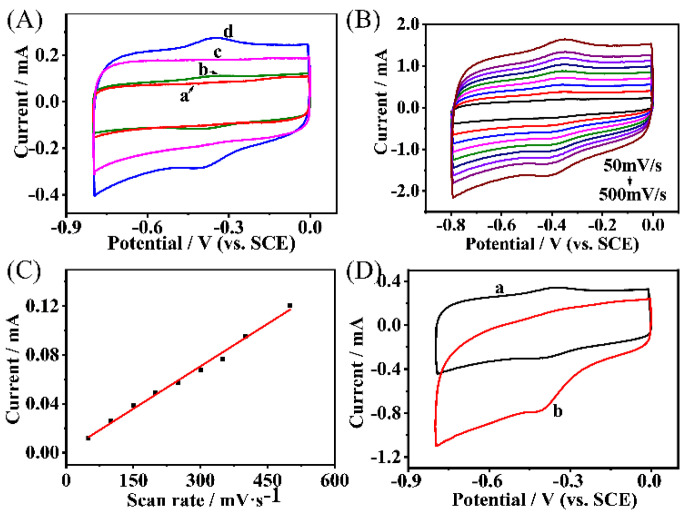
(**A**) Cyclic voltammograms obtained on the modified electrodes in 0.1 M PBS (pH = 7.0) at a scan rate of 100 mV/s: (a) MWNTs/CC; (b) HRP/MWNTs/CC; (c) NH_2_−Hf−BTB−MOL/MWNTs/CC; and (d) HRP/NH_2_−Hf−BTB−MOL/MWNTs/CC. (**B**) Cyclic voltammograms of the HRP/NH_2_−Hf−BTB−MOL/MWNTs/CC electrode in 0.1 M PBS (pH = 7.0) at different scan rates. (**C**) The linear relationship between the peak current and the scan rate. (**D**) Cyclic voltammograms of the HRP/NH_2_−Hf−BTB−MOL/MWNTs/CC electrode in 0.1 M PBS (pH = 7.0) with a scan rate of 100 mV/s: (a) in the absence of H_2_O_2_; and (b) in the presence of 1.5 mM H_2_O_2_.

The voltammetric behaviors of the CMEs, including the MWNTs/CC (Curve a), HRP/MWNTs/CC (Curve b), NH_2_-Hf-BTB-MOL/MWNTs/CC (Curve c), and HRP/NH_2_-Hf-BTB-MOL/MWNTs/CC (Curve d) electrodes, are shown in Figure 1A. Comparing Curve a and Curve c, the charging/discharging currents of the electric double layer were almost doubled, indicating that the electric double-layer (EDL) capacitance of the NH_2_-Hf-BTB-MOL/MWNTs/CC electrode was larger than that of the MWNTs/CC electrodes. Meanwhile, a pair of redox peaks at −0.38 V were observed in both Curve b and Curve d. It was observed that the Faraday current response on the HRP/NH_2_-Hf-BTB-MOL/MWNTs/CC electrode was enhanced much higher than that on HRP/MWNTs/CC electrode. The ratio of Faraday current caused by the redox reaction of HRP over the charging/discharging current of EDL was also improved on the HRP/NH_2_-Hf-BTB-MOL/MWNTs/CC electrode. The results show the facilitated charge transfer between the MWNT/CC network and the HRP molecules immobilized on the 2D MOL through the static interaction between the protonated amino groups and HRP molecules.

Figure 1B shows the cyclic voltammograms with the different scan rates obtained on the HRP/NH_2_-Hf-BTB-MOL/MWNTs/CC electrode. Since the HRP molecules were immobilized on the 2D MOL, the typical voltammetric characteristics of redox adsorption species are presented. The peak potential hardly moved with the scanning rate, and the peak current was found in harmonious proportion to the scan rate (Figure 1C), indicating that the kinetic rate of the HRP redox reaction was pretty reversible. Thus, the effective loading of HRP can be obtained by the following equation [20]:

(1) $$i_{p}=\frac{n^{2}F^{2}A\Gamma v}{4RT}$$

where *i_p_* is the peak current, *n* is electron transfer number (*n* = 1), *F* is the Faraday constant (96,485 C·mol^−1^), *R* is the gas constant (8.314 J·K^−1^·mol^−1^), and *T* is the experimental temperature (298.15 K), *A* is an apparent electrode area, *Γ* is the apparent surface concentration, and *v* is the scan rate (V·s^−1^). From Equation (1), *Γ* was calculated as 3.88 × 10^−10^ mol·cm^−2^ on the HRP/NH_2_-Hf-BTB-MOL/MWNTs/CC electrode, which was 3.3-fold higher than that on HRP/MWNTs/CC electrode (1.17 × 10^−10^ mol·cm^−2^), and 10-fold higher than that in previous reports [21,22,23]. The quantitative results demonstrated that the planar structure of the 2D MOL can improve the effective loading amount of HRP molecules. The voltammetric behavior of the HRP electrocatalytic reduction of H_2_O_2_ obtained on the HRP/NH_2_-Hf-BTB-MOL/MWNTs/CC electrode is shown in Figure 1D.

**Figure 2:**
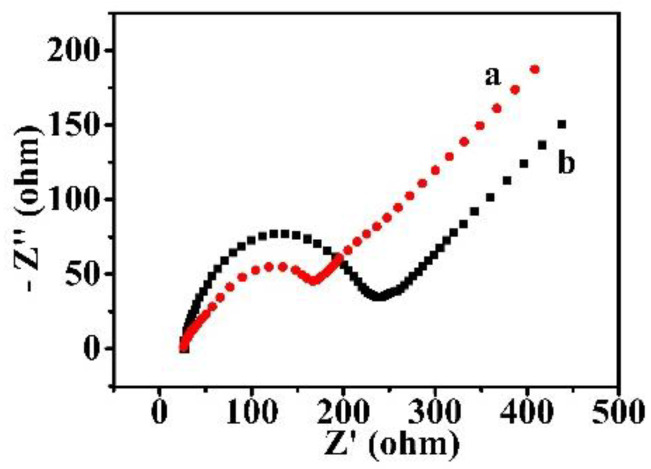
The EIS spectra of (a) HRP/NH_2_-Hf-BTB-MOL/MWNTs/CC; and (b) HRP/MWNTs/CC electrodes in the 0.1 M PBS solution.

To further elucidate the effect of the 2D MOL on the HRP redox reaction, EIS experiments were performed and the results are shown in Figure 2. In the presence of 2D MOL, the charge transfer resistance becomes smaller, indicating that the kinetic rate of electron transfer was improved. Since the 2D MOL has a nanometer thickness and is homogeneously distributed on the MWNTs network, compared with the 3D materials, the tunnel distance of electron transfer between the MWNTs network and the HRP molecules was shortened, and the Faraday current was enhanced in cyclic voltammetry (Figure 1A). However, the EDL capacitance becomes higher in the presence of the 2D MOL in cyclic voltammetry. In general, if the specific capacitance of the electrode material is known, the EDL capacitance can be adopted to estimate the “real” surface or interface area, which is usually higher than the “geometric” area and, in some case, is considered as the active electrode area [24]. Nevertheless, an ultrathin 2D material or even a molecular monolayer (e.g., thiol on gold) could not dramatically change the electrode surface area. Because of the dielectric properties (ε: dielectric constant) of the modified material, the EDL capacity of CMEs usually becomes larger than the bare electrodes or the MWNTs modified electrodes (C = εS/d, here S is the area and d is the thickness). Thus, the real area could not be obtained simply by the EDL capacitance for most CMEs without knowing the dielectric constant and specific capacitance of the modified materials. Fortunately, because the electrochemical sensors work under the steady state, the capacitance affects the response time, but not the detection accuracy. The EIS experiment results (Figure 2) showed that the apparent charge transfer resistance became smaller in the presence of 2D MOL (102 Ω) compared with that in the absence of 2D MOL (170 Ω); meanwhile, the apparent EDL capacitance was obtained as 46.6 μF in the presence of 2D MOL compared with that in the absence of 2D MOL (18.9 μF). The results are in harmonious accordance with those of cyclic voltammetry. It can be concluded that the 2D MOL, as a modified electrode material, cannot only improve the effective loading amount of HRP molecules, but also facilitate electron transfer between the HRP molecules and MWNT/CC conductive network, which predicts an excellent sensing performance, as subsequently shown in Table 1.

**Figure 3:**
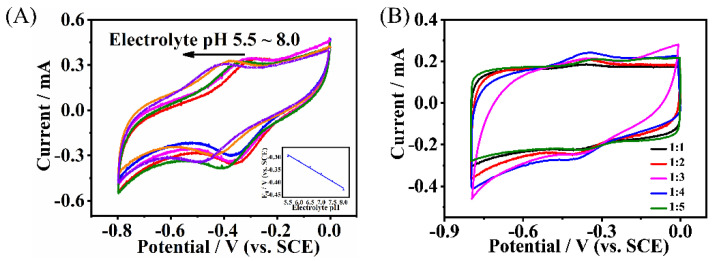
(**A**) Cyclic votammograms obtained on the HRP/NH_2_−Hf−BTB−MOL/MWNTs/CC electrode in 0.1 M PBS at different pH values: 5.5, 6.0, 6.5, 7.0, 7.5, 8.0. Inset: linear relationships of E_θ_ with buffer pH. (**B**) Cyclic votammograms obtained on the HRP/NH_2_−Hf−BTB−MOL/MWNTs/CC electrode with different MOF: HRP ratio in 0.1 M PBS (pH = 7.0). The scan rate is 100 mV/s.

Figure 3A shows the effect of pH on the performance of the HRP/NH_2_-Hf-BTB-MOL/MWNTs/CC electrode in the absence of H_2_O_2_. The peak potential of HRP redox reduction shifted negatively with the increased pH value with a slope of −53.26 mV·pH^−1^, indicating it was a proton-coupled single electron transfer reaction [25]. Considering the biological environment and the Faraday current response, a pH value of 7.0 was chosen to test the sensing performance. To optimize the effective loading amount of HRP on the 2D MOL, a different mass ratio was tried during the pre-loading process when preparing the CME by fixing the concentration of NH_2_-Hf-BTB-MOL at 0.25 mg/mL and the volume at 1 mL. From Figure 3B, it can be seen that the Faraday current response achieves its maximum when the mass ratio of MOF over HRP is 1:4. When the amount of HRP is lower, the adsorption of HRP is not saturated. However, when the amount of HRP is higher, the overlapped HRP can hinder the effective usage of HRP.

**Figure 4:**
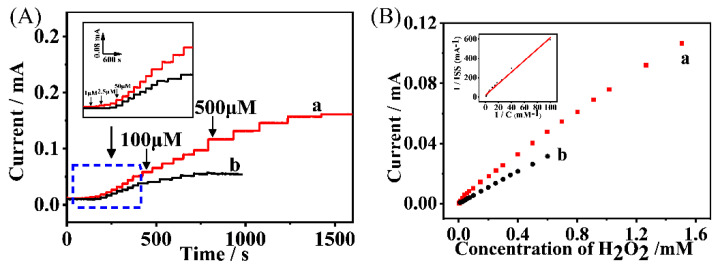
(**A**) The chronoamperometric curves recorded at −0.4 V with the successive injection of H_2_O_2_: (a) the HRP/NH_2_−Hf−BTB−MOL/MWNTs/CC electrode; and (b) the HRP/MWNTs/CC electrode—the insert is the current response at low concentrations of H_2_O_2_. (**B**) The linear relationship between the Faraday current response and the concentration of H_2_O_2_ and the insert is the reciprocal relationship between the Faraday current response and the concentration of H_2_O_2_.

Successive-sample-injection chronoamperometry was carried out to evaluate the sensing performance of the HRP/NH_2_-Hf-BTB-MOL/MWNTs/CC electrode. The applied potential was fixed at −0.4 V and the steady-state Faraday current responses with the injected H_2_O_2_ concentration were recorded, as shown in Figure 4A. The linear relationship between the current response and the H_2_O_2_ concentration ranged from 7.5 μM to 1.5 mM (Figure 4B). Additionally, the sensitivity and detection limit were obtained as 282.4 μA·mM^−1^ ·cm^−2^ and 0.87 μM (S/N = 3). The affinity of H_2_O_2_ on the HRP/NH_2_-Hf-BTB-MOL/MWNTs/CC electrode was also evaluated by the Lineweaver–Burk equation [26]:

(2) $$\frac{1}{I_{\mathrm{ss}}}=\frac{1}{I_{\max}}+\frac{K_{m}{}^{\mathrm{app}}}{I_{\max}C}$$

where K_m_^app^ is the Michaelis–Menten constant, I_ss_ is the steady-state current after H_2_O_2_ injection, C is the concentration of H_2_O_2_, and I_max_ is the limiting enzyme-catalytic current when H_2_O_2_ is saturated. The K_m_^app^ was obtained as 0.22 mM from the linear relationship between 1/I_ss_ and 1/C (as the insert in Figure 4B), indicating the good affinity of H_2_O_2_ on the HRP/NH_2_-Hf-BTB-MOL/MWNTs/CC electrode. Compared with previous reports, this 2D-MOL supported enzyme biosensor presents high sensitivity and high affinity, as listed in Table 1.

**Table 1:** Parameters of the reportedly modified HRP electrodes.

HRP Electrodes	Sensitivity (μA·mM^−1^·cm^−2^)	Linear Range (μM)	LOD (μM)	K_m_ (μM)	Reference
HRP/GO-Co_3_O_4_-Nafion/GCE	18.7 ± 0.5	1000–30,000	2000	—	[27]
PEDOT/PB/PPyBA/HRP	1.15	100–700	30	-	[28]
HRP/chitosan-NiFe_2_O_4_/GCE	0.314	300–1200	14	1400	[29]
HRP/TBA−COOH−IL/MWCNT/GCE	160.6	20–4300	6	—	[30]
[(ZIF-8@HRP/GO)/(GO-PEI)]_4_/ITO	—	20–6000	3.4	9250	[16]
HRP/SiO_2_/BSA/Au	—	8–3720	2.0	2300	[31]
HRP/GO/GCE	120	2–500	1.6	—	[32]
BPT/AuNPs/graphene/HRP/Au	—	5–2500	1.5	—	[33]
HRP/PDA-MNPs/(l-Arg/Tb)/GCE	—	0.5–30	0.23	—	[34]
NH_2_-MIL-53(Fe)/HRP/MWNTs/GCE	—	0.1–1, 1–600	0.028	—	[2]
HRP/MWNTs/CC	185.2	50–600	5.8	330	This work
HRP/NH_2_-Hf-BTB-MOL/MWNTs/CC	282.4	7.5–1500	0.87	220	This work

**Figure 5:**
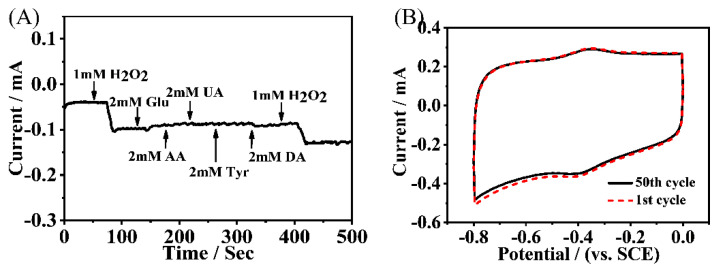
(**A**) HRP/NH_2_-Hf-BTB-MOL/MWNTs/CC amperometric response to 2 mM different interfering substances (−0.4 V vs. SCE); and (**B**) Stability of HRP/NH_2_-Hf-BTB-MOL/MWNTs/CC in 0.1 M PBS (pH = 7.0) containing 0.5 mM H_2_O_2_.

The interference performance caused by the electroactive substrates in the human body such as glucose, uric acid, ascorbic acid, lysine, dopamine, etc., were investigated [17,35]. The concentration of each interference substance used was 2 mM, i.e., 2-fold of that of H_2_O_2_ (1 mM) in the experiment. As shown in Figure 5A, these substrates did not affect the electrochemical detection of H_2_O_2_ because of the specific reduction of HRP to H_2_O_2_. Moreover, five groups of parallel experiments were performed in the presence of 0.5 mM H_2_O_2_ and the relative standard deviation of the Faraday current was 1.36%, indicating the good reproducibility of the preparation protocols of the HRP/NH_2_-Hf-BTB-MOL/MWNTs/CC electrode. Moreover, after 50 cycles, the Faraday current was kept at 91.49% of the original (Figure 5B), which indicated the good stability of this biosensor. All the above results show that the 2D MOL materials have significant potential in the applications of electrochemical biosensors.

## 3. Materials and Methods

### 3.1. Chemicals and Materials

Carbon cloth (CC) was purchased from Carbon Energy Technology Co., Ltd. (Taiwan, China). Multi-walled carbon nanotubes (MWNTs) was purchased from Nanjing XFNANO Materials Technology Co., Ltd. (Nanjing, China). Nafion (5 wt%) was purchased from Guangzhou Lige Technology Co., Ltd. (Guangzhou, China). Ready-to-use PBS powder was purchased from Shanghai Sangon Biotech Co., Ltd. (Shanghai, China). Horseradish peroxidase (HRP), uric acid (UA), ascorbic acid (AA), glucose (Glu), dopamine (DA), and tyrosine (Tyr) were obtained from Aladdin Biochemical Technology Co., Ltd. (Shanghai, China). Aqueous solutions used in the experiments were prepared with deionized water (18.2 MΩ·cm, Milli-Q, Millipore Co.). Nitrogen gas (N_2_, 99.999%) was purchased from Linde Gas Co.

### 3.2. The Fabrication of HRP/NH_2_-Hf-BTB-MOL Modified Electrodes

The 2D MOL was a gift from Dr. Huihui Hu who is with Prof. Cheng Wang’ group at Xiamen University [36]. NH_2_-Hf-BTB-MOL was prepared by grafting an amino-terminated group by stirring 30 mL Hf-BTB-MOL (60 mM based on Hf in H_2_O) and 50 mg ethanolamine phosphate at 60 °C overnight. HRP molecules were pre-loaded on the newly prepared NH_2_-Hf-BTB-MOL by ultrasonic dispersion (Ultrasonic cleaner, KQ5200DE, Kunshan Ultrasonic Instrument Co., Ltd., Kunshan, China) of their mixture with different mass ratios in a 400 μL PBS buffer (0.1 M, pH = 7.0), and a subsequent constant-temperature oscillation (constant temperature water bath oscillator, SHA-C, Changzhou Xiangtian, Changzhou, China) for 1 h at 4 °C.

**Figure 6:**
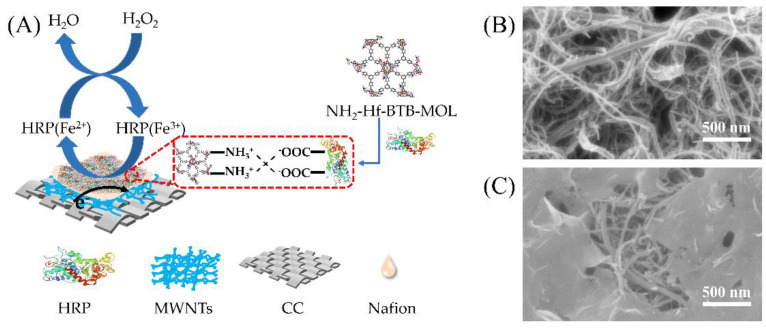
(**A**) Schematic diagram for H_2_O_2_ detection on the HRP/NH_2_−Hf−BTB−MOL/MWNTs/CC biosensor; and (**B**,**C**) the SEM images of the MWNTs/CC and the NH_2_−Hf−BTB−MOL/MWNTs/CC modified electrodes, respectively.

Carbon cloth (CC) was cut into pieces of 1 cm × 1 cm, alternately washed with acetone, ethanol, and water for 5 min, and dried on a heating plate. Then, 1 mg/mL MWNTs was dispersed ultrasonically in ethanol solution for 30 min. Subsequently, a 20 μL MWNTs suspension was dropped onto the CC piece to form a MWNTs/CC current collection network. After that, 20 μL HRP/NH_2_-Hf-BTB-MOL suspension was drop-coated on the MWNTs/CC and dried at room temperature. Finally, 20 μL Nafion solution (5 wt%) was dropped to solidify the coatings. The prepared HRP/NH_2_-Hf-BTB-MOL/MWNTs/CC electrode was stored in a refrigerator at 4 °C for use. For comparison, the MWNTs/CC, HRP/MWNTs/CC, and NH_2_-Hf-BTB-MOL/MWNTs/CC electrodes were also prepared with the same protocols as schemed in Figure 6A. The SEM images of the MWNTs/CC and the NH_2_-Hf-BTB-MOL/MWNTs/CC-modified electrodes are shown in Figure 6B,C.

### 3.3. Electrochemical Measurements

Electrochemical experiments were performed with a CHI 850D electrochemical workstation (Shanghai Chenhua Co., Shanghai, China). Electrochemical impedance spectroscopy (EIS) experiments were performed with Autolab electrochemical workstation (Ω Metrohm Co., Herisau, Switzerland). A three-electrode system was adopted, where a saturated calomel electrode (SCE) and a platinum (Pt) wire electrode served as the reference electrode and counter electrode, respectively. The solution was bubbled gently by high-purity N_2_ to degas oxygen before experiment.

## 4. Conclusions

Employing a 2D NH_2_-Hf-BTB-MOL material as the support for HRP, we developed an electrochemical biosensor for the rapid, reliable, and precise detection of H_2_O_2_. Both the direct redox behaviors of HRP molecules and the electrocatalytic reduction of H_2_O_2_ were observed on the HRP/NH_2_-Hf-BTB-MOL/MWNTs/CC electrode. By optimizing the pH value and effective loading amount of HRP, we found that the electrochemical enzyme sensor has excellent sensitivity, selectivity, affinity, and stability to H_2_O_2_. All the results indicate that the 2D MOLs have significant potential in the applications of electrochemical enzyme sensors because the ultrathin 2D MOLs cannot only facilitate the electron transfer, but also improve the effective loading amount of the enzyme molecules.

## Data Availability

The authors confirm that the data supporting the findings of this study are available within the article and its supplementary materials.

## Sample Availability

Samples of the compounds are available from the authors upon reasonable request.
